# Supplementary material for: A Human-Immune-System (HIS) humanized mouse model (DRAGA: HLA-A2. HLA-DR4. Rag1 KO.IL-2Rγc KO. NOD) for COVID-19
Source: bioRxiv. 2021 Jan 29:2020.08.19.251249. Originally published 2020 Aug 20. Preprint. [Version 3] doi: 10.1101/2020.08.19.251249 (PMC7444284; doi:10.1101/2020.08.19.251249)
Supplement: 1 [file NIHPP2020.08.19.251249-supplement-1.pdf]

bioRxiv preprint doi: https://doi.org/10.1101/2020.08.19.251249; this version posted January 29, 2021. The copyright holder for this preprint (which was not certified by peer review) is the author/funder. This article is a US Government work. It is not subject to copyright under 17 USC 105 and is also made available for use under a CC0 license.

Table S1. Human immune parameters of HIS-DRAGA mice

			**Peripheral blood**				
**Mouse***	**Gender**	**Time post-infusion with human stem cells**	**% human B cells (CD19⁺)**	**% human T cells (CD3⁺)**	**SARS-CoV-2 challenge dose (pfu)**	**Euthanasia (days post-infection)**	**Cord blood HLA haplotype^**
M#1	M	21 weeks	4.5	13.5	$2.8x10^3$	1(died)	A
F#1	F	21 weeks	48.6	9.3	$2.8x10^3$	14	A
F#2	F	21 weeks	21	33.4	$2.8x10^4$	14	A
F#3	F	16 weeks	9.0	2.5	$1x10^3$	25	B
F#4	F	16 weeks	37.0	4.7	$1x10^3$	25	B
F#5	F	16 weeks	23.6	5.0	$1x10^3$	25	B
F#6	F	16 weeks	0.6	12.5	$1x10^3$	25	B
F#7	F	30 weeks	5.9	42.6	$1x10^3$	25	A
F#8	F	24 weeks	16.1	37.8	$1x10^3$	25	C
F#9	F	16 weeks	21.5	8.0	$1x10^3$	25	B
F#10	F	24 weeks	6.0	23.8	$1x10^3$	25	C
M#2	M	30 weeks	8.3	9.3	$1x10^3$	3	A
M#3	M	16 weeks	5.7	15.0	$1x10^3$	3	B
M#4	M	16 weeks	2.0	51.1	$1x10^3$	3	B
F#11	F	16 weeks	6.1	25.4	$1x10^3$	3	B
F#12	F	24 weeks	7.0	8.8	$1x10^3$	3	C
F#13	F	24 weeks	0.6	26.8	$1x10^3$	3	C
a	M	20 weeks	18.9	28.3	----	----	D
b	M	20 weeks	0.9	41.4	----	----	D
c	F	24 weeks	52.5	17.5	----	----	D
d	M	24 weeks	7.4	16.6	----	----	D
e	M	24 weeks	1.0	22.1	----	----	D
f	M	24 weeks	1.2	44.7	----	----	D
g	M	24 weeks	17.6	38.3	----	----	D
h	F	20 weeks	35.2	5.0	----	----	E
i	M	26 weeks	4.9	26.1	----	----	E
j	M	26 weeks	21.9	30.7	----	----	E

Lungs from uninfected mice a-j were pooled to assess human ACE2 mRNA and protein expression.

^A: A02:01/A24:02/B13:02/B44:02/DR01:01/DR04:01

^B: A01:01/A02:01/B08:01/B15:01/DR03:01/DR04:01

^C: A02:01/A24:02/B44:02/B52:01/DR04:01/DR11:04

^D: A02:01/A02:01/B18:01/B44:02/DR04:01/DR11:04

^E: 02:01/A02:01/B08:01/B27:05/DR03:01/DR04:01

bioRxiv preprint doi: https://doi.org/10.1101/2020.08.19.251249; this version posted January 29, 2021. The copyright holder for this preprint (which was not certified by peer review) is the author/funder. This article is a US Government work. It is not subject to copyright under 17 USC 105 and is also made available for use under a CC0 license.

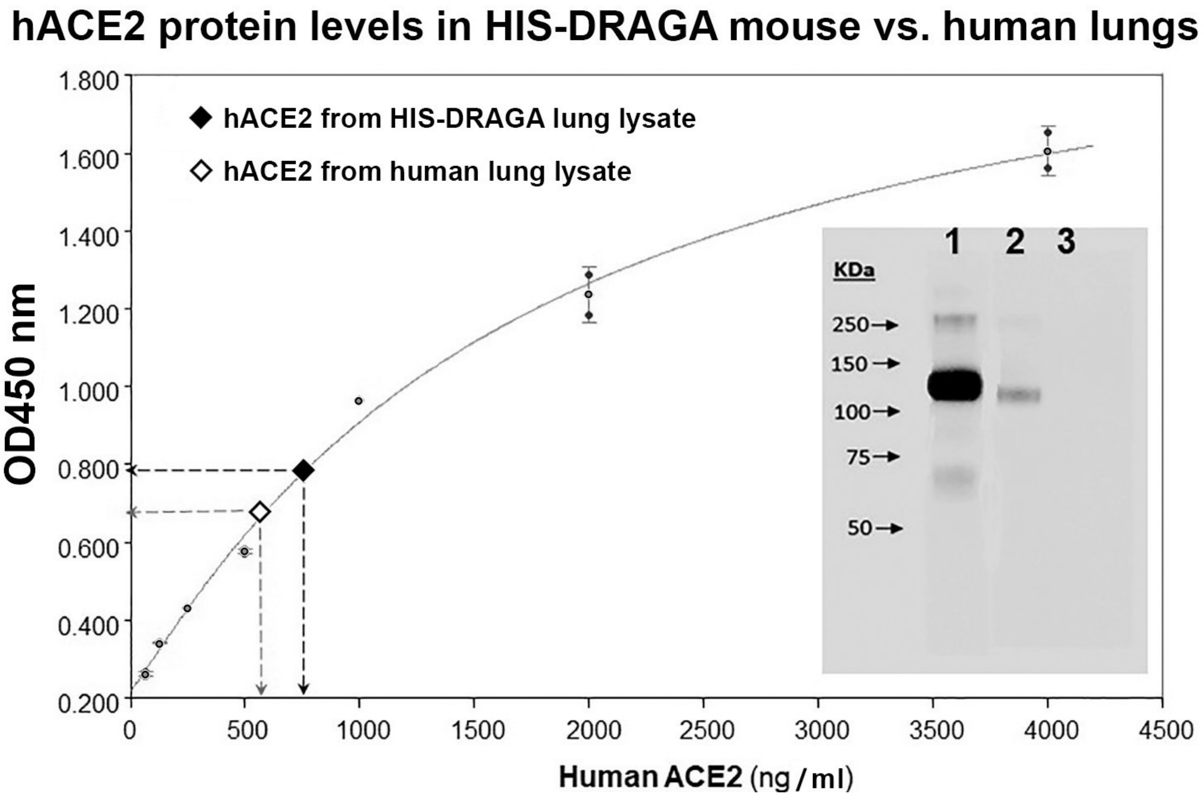

Sample	Concentrated immunoprecipitate	[hACE2] from ELISA	dilution	[hACE2] in total lung protein
2 mg Human lung protein	52 µl	655 ng / ml	1:100	1.703 µg / mg
9 mg HIS-DRAGA lung protein	52 µl	757 ng / ml	1:50	0.219 µg / mg
9 mg DRAGA lung protein	52 µl	ND*	1:50	ND*

*ND = Not Detectable

Ratio [hACE2] in human lung / [hACE2] in HIS-DRAGA mouse lung (ELISA) = 7.8

Fig. S1

Figure S1. Quantification of hACE2 protein in HIS-DRAGA lungs. Human ACE2 levels in immunoprecipitates obtained from non-infected HIS-DRAGA and human lung lysates using S1(RBD)-mFc□2a protein + rat anti-mouse IgG2a-magnetic beads were quantified by ELISA. Of note, the OD450nm values for protein immunoprecipitated from a pool of 10 non-infected, non-HIS-humanized DRAGA mouse lung lysates (negative control) fell below the limit of detection (OD450nm <0.05). ***Insert*** shows Western blot detection of hACE2 protein in the concentrated immunoprecipitates probed with a mouse monoclonal anti-human ACE2 IgG followed by goat anti-mouse IgG-HRP with ECL detection. *Lane 1*, human lung immunoprecipitate; *lane 2*, HIS-DRAGA mouse lungs immunoprecipitate; *lane 3*, DRAGA mouse lungs immunoprecipitate (note this sample did not contain detectable hACE2). Lower panel shows the experimental conditions for immunoprecipitation of hACE2, quantification by ELISA, and the ratio of hACE2 in human versus HIS-DRAGA mouse lung samples.

bioRxiv preprint doi: https://doi.org/10.1101/2020.08.19.251249; this version posted January 29, 2021. The copyright holder for this preprint (which was not certified by peer review) is the author/funder. This article is a US Government work. It is not subject to copyright under 17 USC 105 and is also made available for use under a CC0 license.

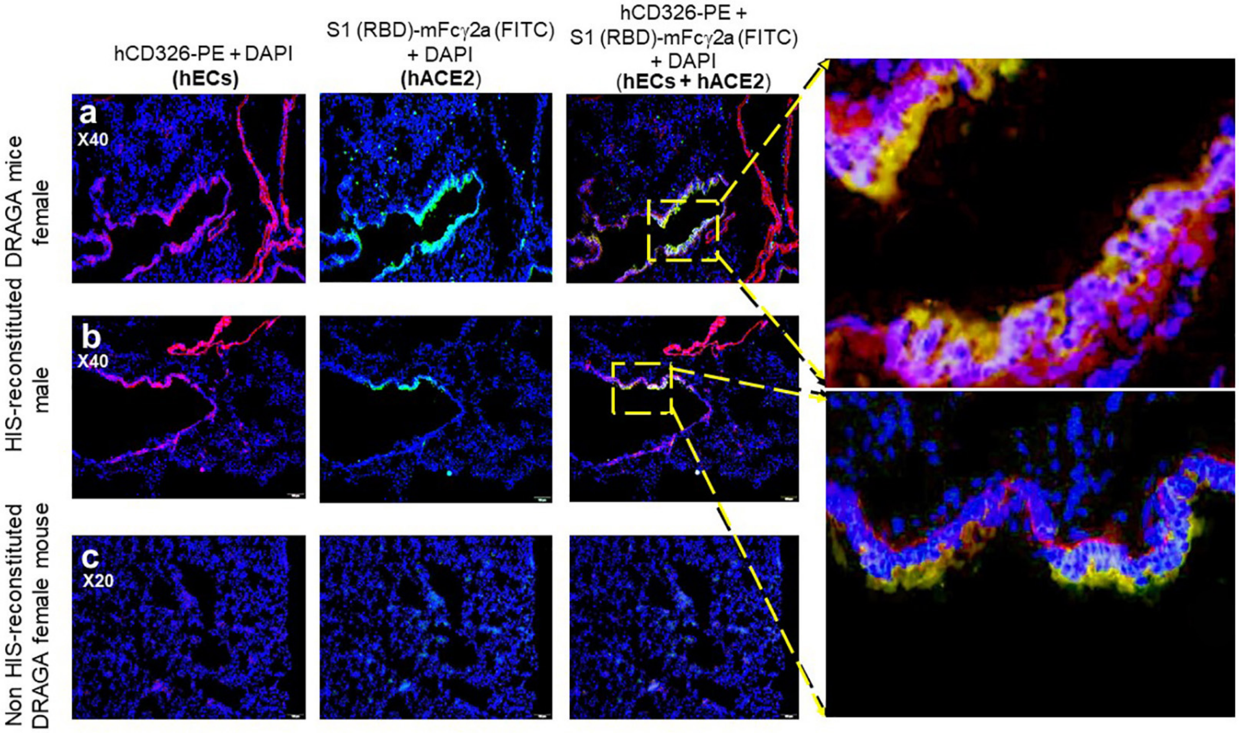

Fig. S2

Figure S2. Co-localization of hACE2 receptor with hCD326⁺ alveolar ECs in a non-infected HIS-DRAGA mouse. a,**b.** Co-localization of hACE2 with alveolar hCD326⁺ ECs (orange) revealed by co-staining of S1(RBD)-mFc□2a protein + goat anti-mouse IgG-FITC (green) and anti-hCD326-PE (red) in representative HIS-DRAGA female and male mice. **c.** Lack of hCD326⁺ ECs and negligible binding of S1(RBD)-mFc□2a protein to a representative lung section from a non-HIS-reconstituted DRAGA female mouse.

bioRxiv preprint doi: https://doi.org/10.1101/2020.08.19.251249; this version posted January 29, 2021. The copyright holder for this preprint (which was not certified by peer review) is the author/funder. This article is a US Government work. It is not subject to copyright under 17 USC 105 and is also made available for use under a CC0 license.

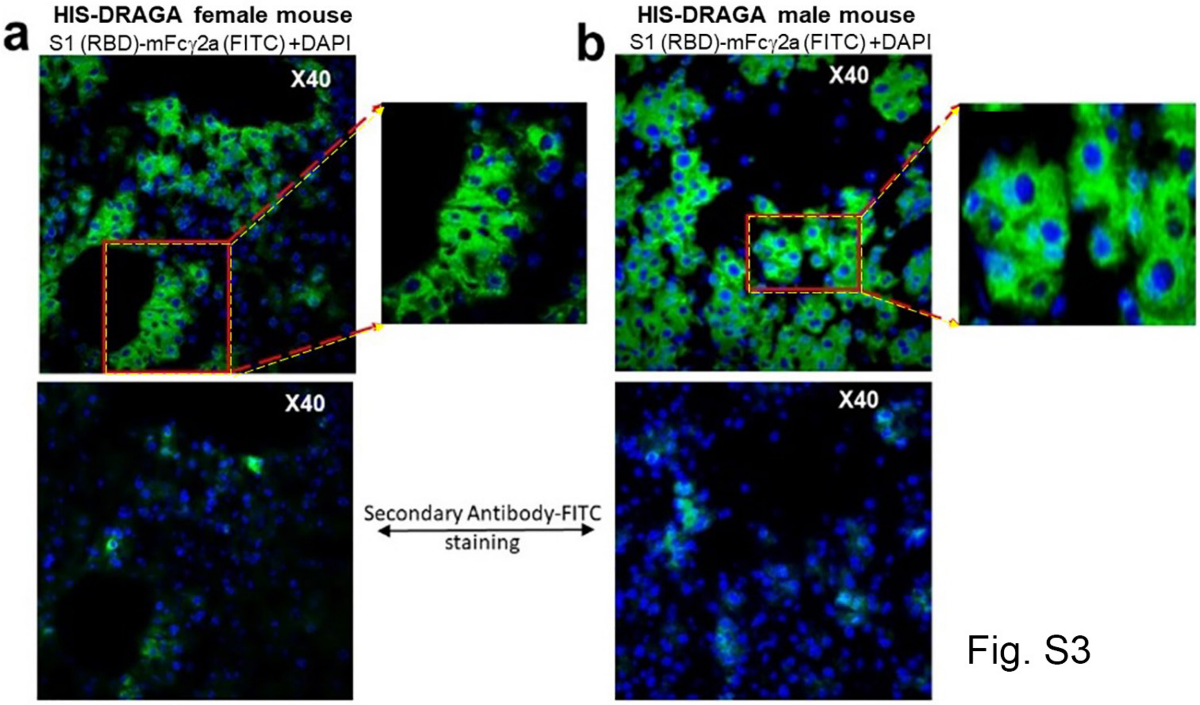

Fig. S3

Figure S3. Binding of SARS-CoV-2 S1(RBD) protein to the endothelium of liver cholangiocytes in non-infected HIS-DRAGA mice. S1(RBD) binding to the liver cholangiocytes from representative non-infected HIS-DRAGA female (*panel a)* and male *(panel b)* mice. Merged images and enlargements show binding of S1(RBD)-mFc□2a revealed by a goat anti-mouse IgG-FITC conjugate (green) and nuclei (DAPI, blue). *Lower* panels, representative images showing minimal background binding of the goat anti-mouse IgG-FITC secondary antibody (green) and nuclei (DAPI, blue) in tissues from the same mice in panels a and b.

bioRxiv preprint doi: https://doi.org/10.1101/2020.08.19.251249; this version posted January 29, 2021. The copyright holder for this preprint (which was not certified by peer review) is the author/funder. This article is a US Government work. It is not subject to copyright under 17 USC 105 and is also made available for use under a CC0 license.

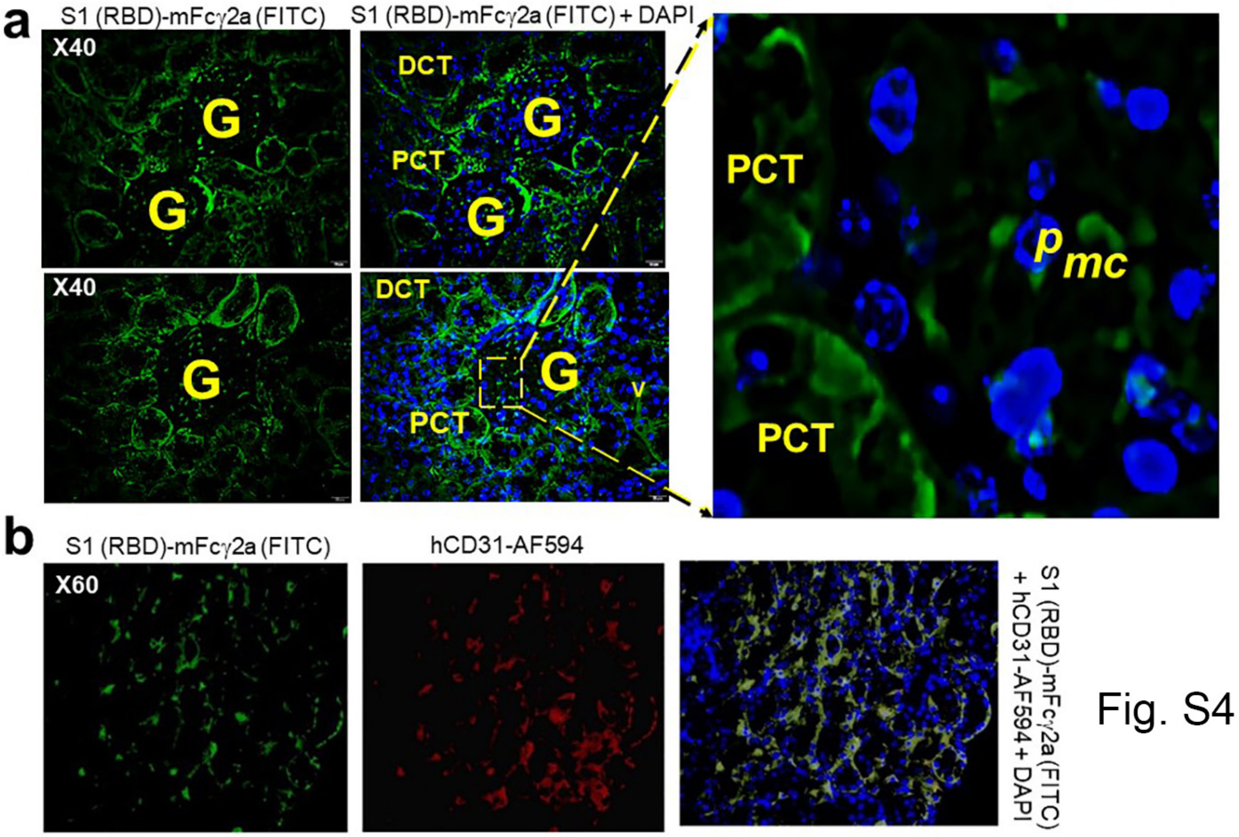

Figure S4. Binding of SARS-CoV-2 S1 (RBD) protein to kidney epi/endothelia of infected HIS-DRAGA mice. a. Sections of renal cortex from HIS-DRAGA survivors #F1 (upper panels) and #F2 (lower panels) of SARS-CoV-2 infection with $2.8x10^4$ pfu and $2.8x10^3$ pfu, respectively, at the experimental endpoint (14 dpi). Sections were co-stained with DAPI (blue) and S1(RBD)-mFc□2a protein + goat anti-mouse IgG-FITC (green). Shown is the S1(RBD) protein bound to the epithelium layer of proximal convoluted tubules (PCT) and distal convoluted tubules (DCT) surounding the glomeruli (G). Enlargement of a peripheral glomerular area shows nuclei (blue) of podocytes (p) and the endothelia (green) of glomerular microcapilaries (mc) in close proximity to the podocytes (green). **b.** Expression of hACE2 revealed by S1(RBD)-mFc□2a binding (green) on kidney epithelial cells labeled with an anti-hCD31-AF594 antibody (red).

bioRxiv preprint doi: https://doi.org/10.1101/2020.08.19.251249; this version posted January 29, 2021. The copyright holder for this preprint (which was not certified by peer review) is the author/funder. This article is a US Government work. It is not subject to copyright under 17 USC 105 and is also made available for use under a CC0 license.

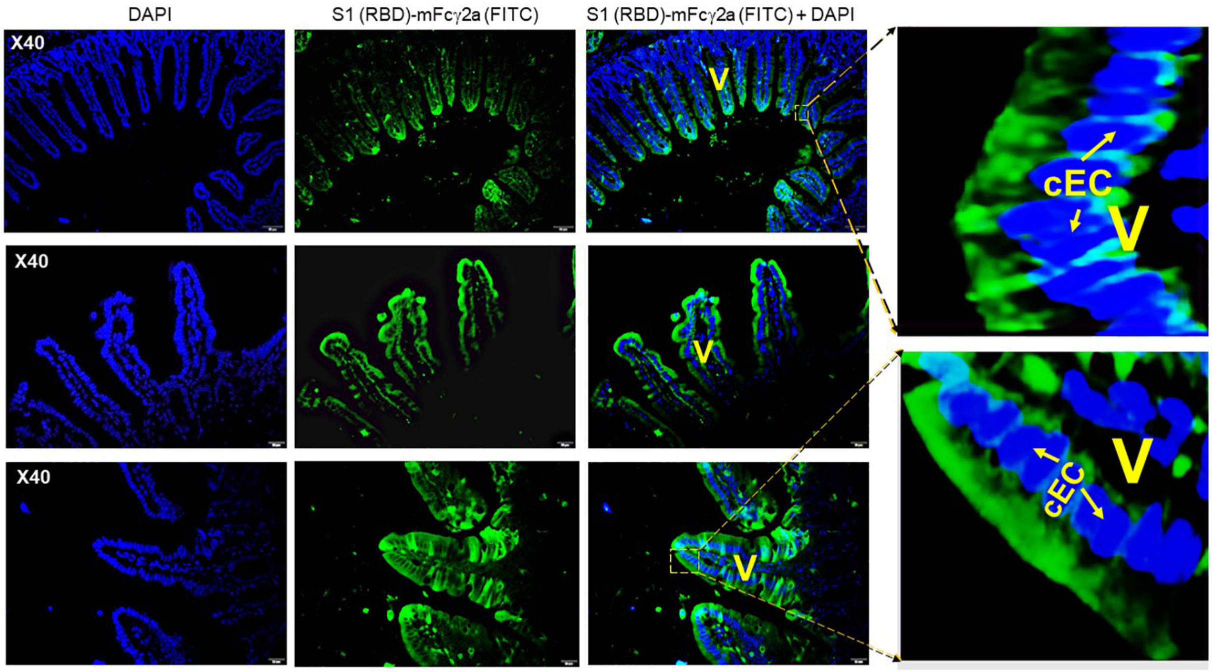

Fig. S5

Figure S5. Binding of SARS-CoV-2 S1(RBD) protein to intestinal epithelia of infected HIS-DRAGA mice. Tissue sections from the small intestine of HIS-DRAGA mouse #F1 (*upper and middle panels*) and #F2 (*lower panel*) survivors of SARS-CoV-2 infection with 2.8x10^4 pfu and 2.8x10^3 pfu, respectively, at the experimental endpoint (14 dpi). Sections were co-stained with DAPI (nuclei, blue) and S1(RBD)-mFc□2a protein+ goat anti-mouse IgG-FITC (green). Shown is the S1(RBD) protein bound to the columnar epithelial cells (cEC) of the absorptive intestinal villi (V).

bioRxiv preprint doi: https://doi.org/10.1101/2020.08.19.251249; this version posted January 29, 2021. The copyright holder for this preprint (which was not certified by peer review) is the author/funder. This article is a US Government work. It is not subject to copyright under 17 USC 105 and is also made available for use under a CC0 license.

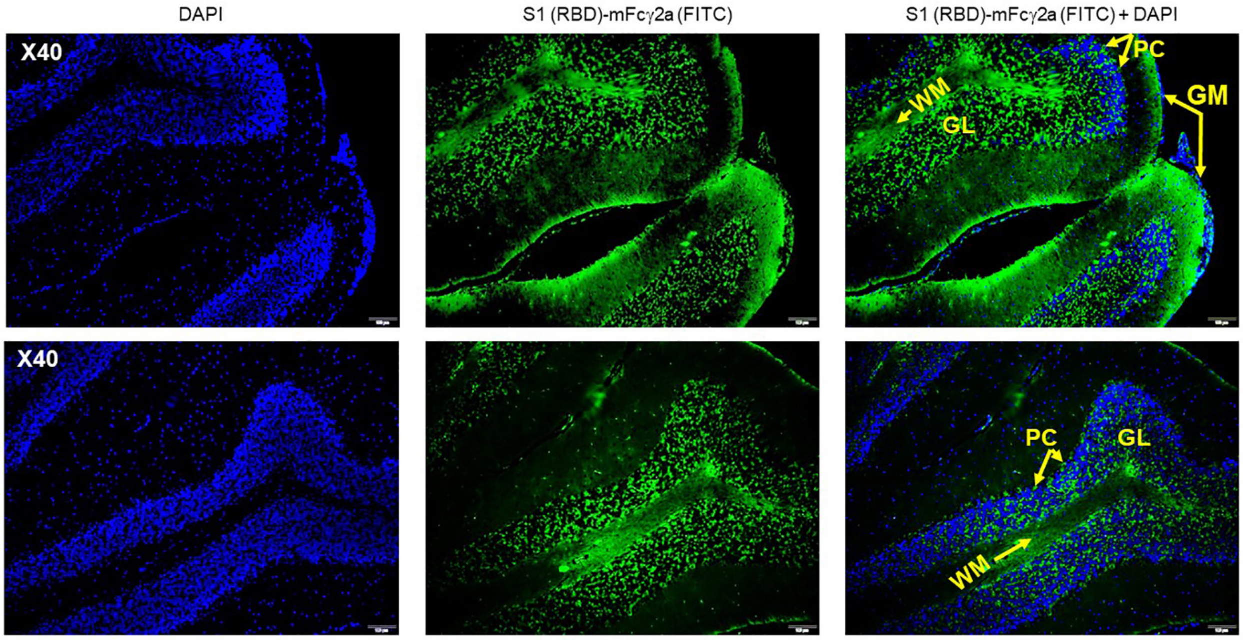

Fig. S6

Figure S6. Binding of SARS-CoV-2 S1(RBD) protein to the brain epithelia in infected HIS-DRAGA mice. Tissue sections of cerebellum cortex from HIS-DRAGA mice #F1 (*upper panel*) and #F2 (*lower panel*) survivors of SARS-CoV-2 infection with $2.8x10^4$ pfu and $2.8x10^3$ pfu, respectively, at the experimental endpoint (14 dpi) co-stained with DAPI (nuclei, blue) and S1(RBD)-mFc□2a protein + goat anti-mouse IgG-FITC (green). Shown is the S1(RBD) protein bound to the white matter (WM), granular layer (GL), and Purkinje cells (PC), but not to the outer neuronal layer in the grey matter (GM).

bioRxiv preprint doi: https://doi.org/10.1101/2020.08.19.251249; this version posted January 29, 2021. The copyright holder for this preprint (which was not certified by peer review) is the author/funder. This article is a US Government work. It is not subject to copyright under 17 USC 105 and is also made available for use under a CC0 license.

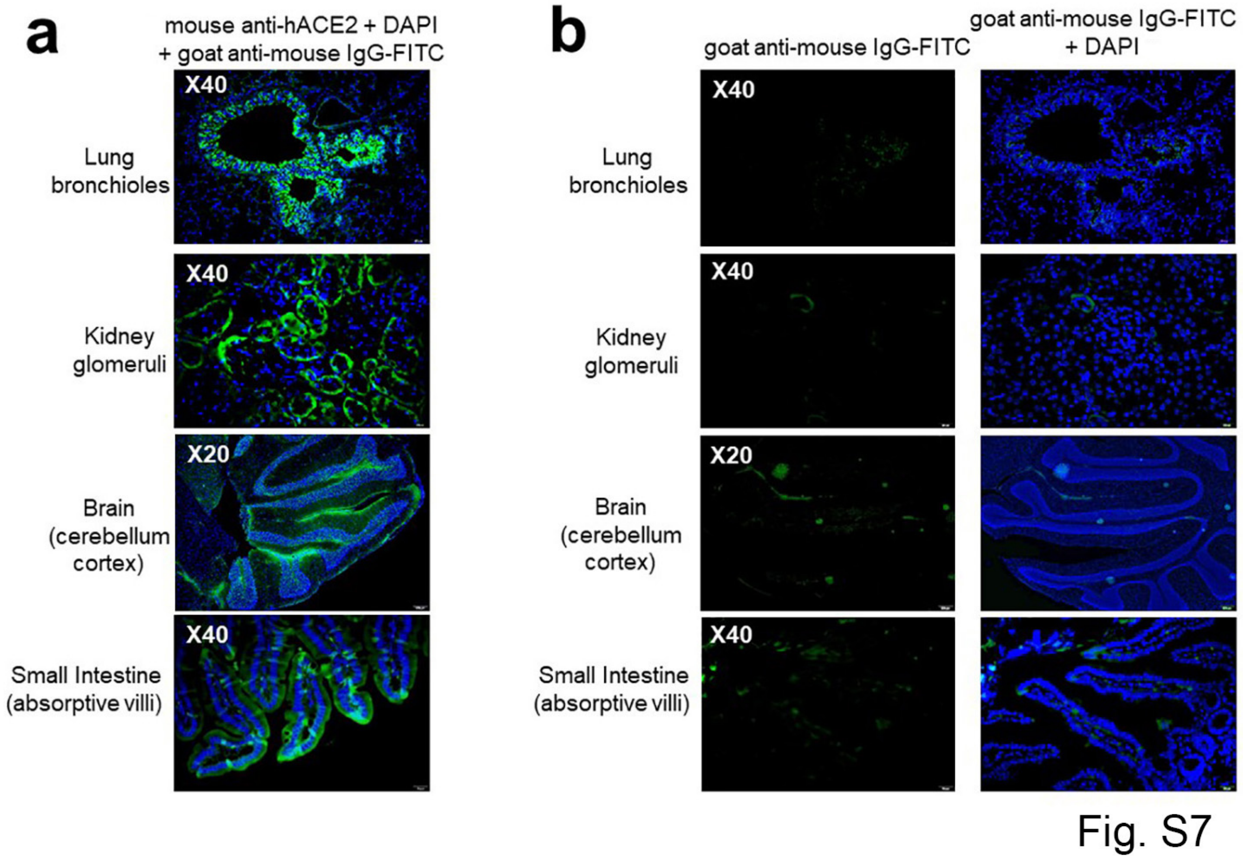

Figure S7. Identification of hACE2 on tissue sections from organs of a non-infected HIS-DRAGA mouse, detected with an anti-hACE2 specific antibody. a. Merged images of tissue sections from a representative non-infected HIS-DRAGA mouse stained with mouse anti-hACE2 followed by goat anti-mouse IgG-FITC (green) and DAPI (blue). **b.** Minimal background binding of the secondary antibody (goat anti-mouse IgG-FITC) (left) overlapped with DAPI staining (right) of tissue sections adjacent to those shown in *panel a*.

bioRxiv preprint doi: https://doi.org/10.1101/2020.08.19.251249; this version posted January 29, 2021. The copyright holder for this preprint (which was not certified by peer review) is the author/funder. This article is a US Government work. It is not subject to copyright under 17 USC 105 and is also made available for use under a CC0 license.

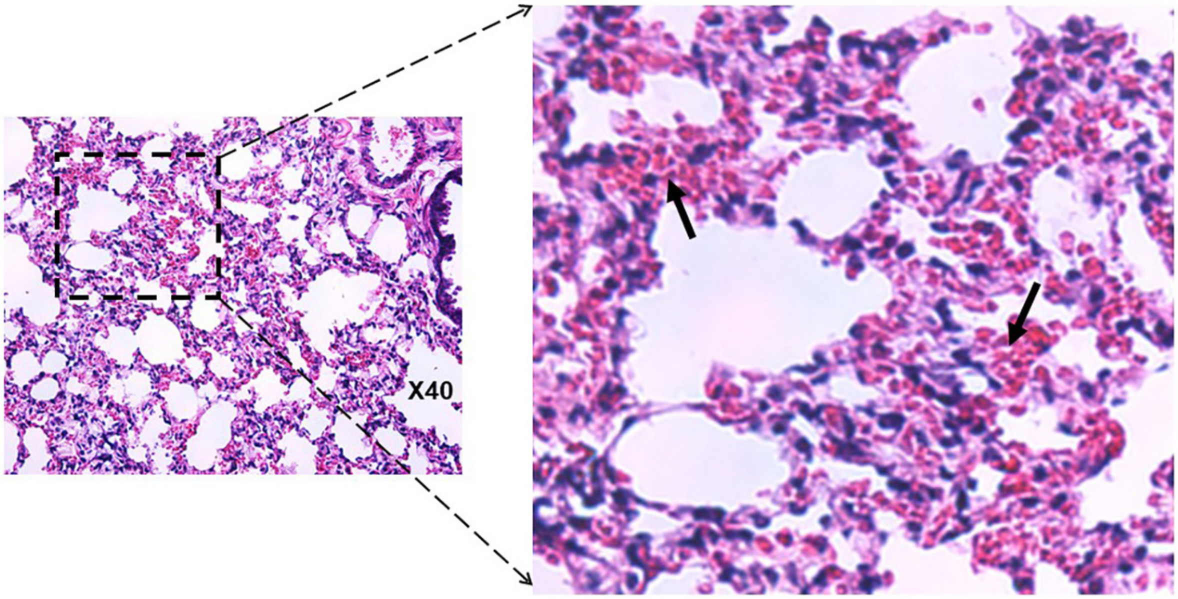

Fig. S8

Figure S8. Lung pathology of a HIS-DRAGA mouse recovering from SARS-CoV-2 infection. Representative H&E-stained lung section from HIS-DRAGA mouse #F2 that recovered its initial body weight at 9 days after infection with SARS-CoV-2 at 2.8x10^3 pfu. Interstitial and intra-alveolar infiltrates are indicated by arrows.

bioRxiv preprint doi: https://doi.org/10.1101/2020.08.19.251249; this version posted January 29, 2021. The copyright holder for this preprint (which was not certified by peer review) is the author/funder. This article is a US Government work. It is not subject to copyright under 17 USC 105 and is also made available for use under a CC0 license.

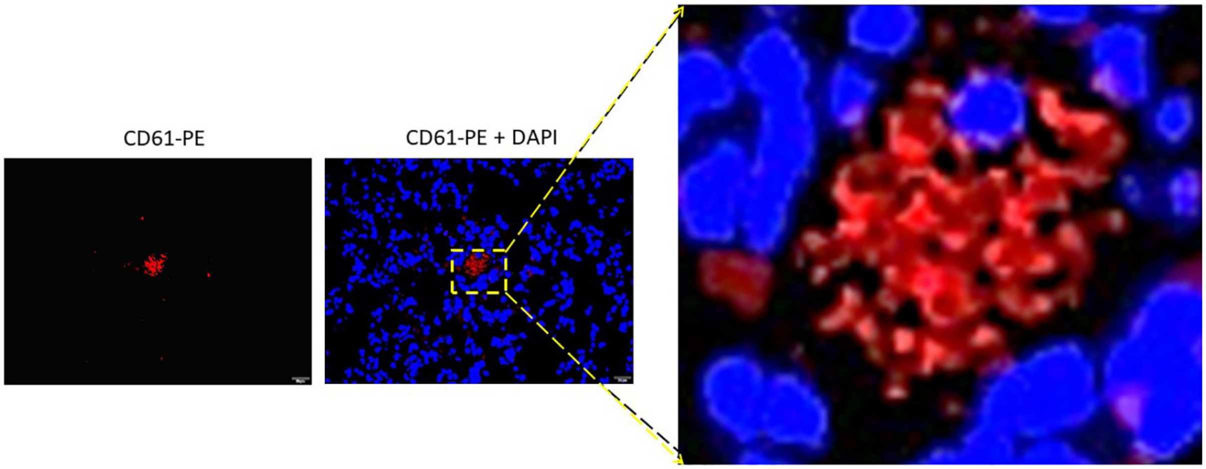

Fig. S9

Figure S9. CD61⁺ intra-alveolar microthrombi in a SARS-CoV-2 infected HIS DRAGA mouse. Intra-alveolar microthrombi in lung section from a HIS-DRAGA mouse infected with 2.8×10^4 pfu of SARS-CoV-2, which had not recovered its initial body weight by 14 dpi. *Left panel*, staining with anti-CD61-PE (red). *Right panel* with enlargement: merged anti-CD61-PE co-staining with DAPI (blue). The non-nucleated $CD61^+$ cluster indicates this is a platelet micro-thrombus.

bioRxiv preprint doi: https://doi.org/10.1101/2020.08.19.251249; this version posted January 29, 2021. The copyright holder for this preprint (which was not certified by peer review) is the author/funder. This article is a US Government work. It is not subject to copyright under 17 USC 105 and is also made available for use under a CC0 license.

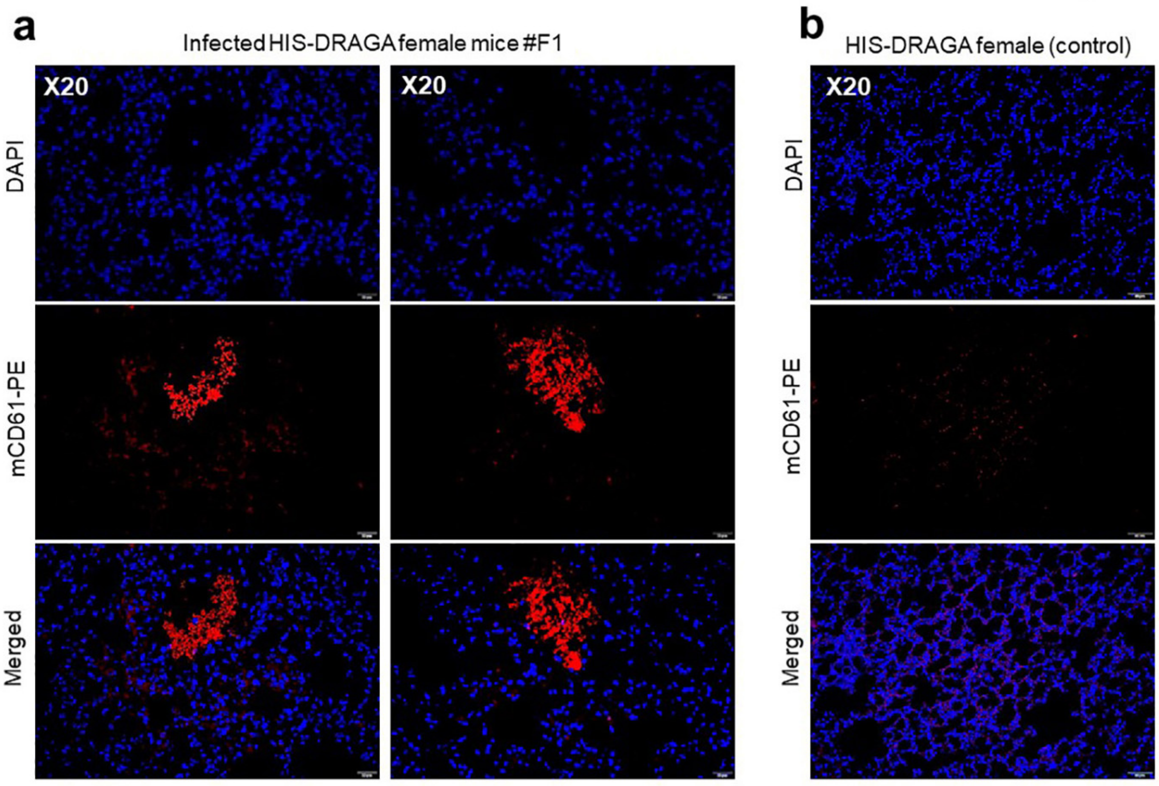

Fig. S10

Figure S10. Large intra-alveolar thrombi in a SARS-CoV-2 infected HIS-DRAGA mouse. a. Representative lung section from HIS-DRAGA mouse #F1 infected with $2.8x10^4$ pfu, which had not recovered its initial body weight by 14 dpi. Co-staining with anti-CD61-PE (red) + DAPI (blue). **b.** Representative lung section from a non-infected HIS-DRAGA mouse stained as in panel a, with no evidence of intra-alveolar thrombi.

bioRxiv preprint doi: https://doi.org/10.1101/2020.08.19.251249; this version posted January 29, 2021. The copyright holder for this preprint (which was not certified by peer review) is the author/funder. This article is a US Government work. It is not subject to copyright under 17 USC 105 and is also made available for use under a CC0 license.

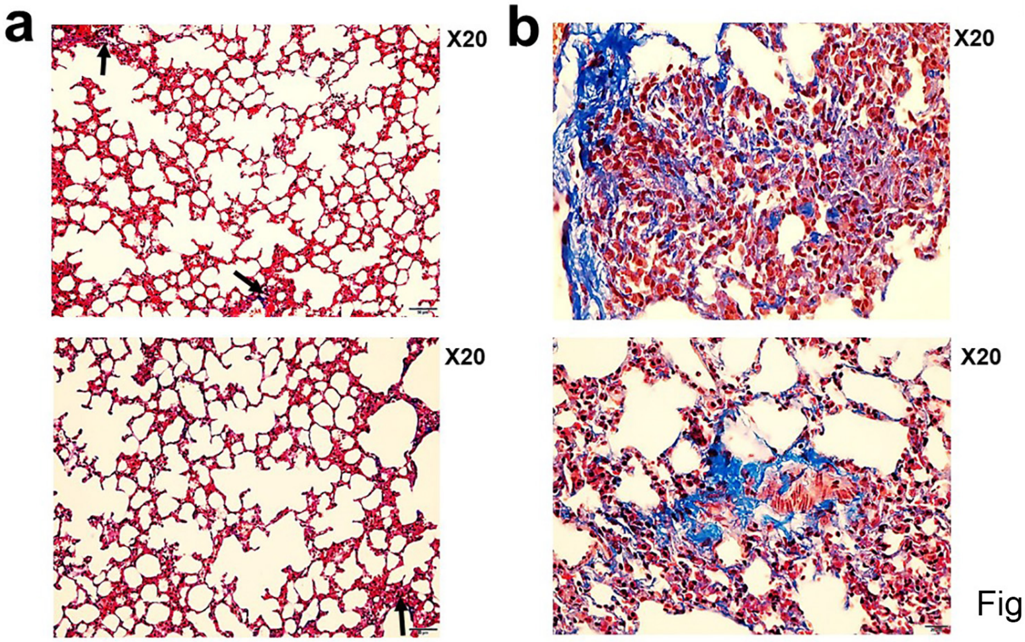

Fig. S11

Figure S11. Pulmonary sequelae in SARS-CoV-2 infected DRAGA mice. a. Masson's Trichrome staining of lung sections from HIS-DRAGA mice #F3 (upper panel) and #F4 (lower panel) infected with 10^3 pfu SARS-CoV-2, which recovered their initial body weights by 9 and 25 dpi, respectively. Shown are small peri-alveolar infiltrates building collagen fibers (blue, arrows). **b.** Masson's Trichrome staining of lung sections from HIS-DRAGA mice #F5 (*upper panel*) and #F6 (*lower panel*) infected with 10^3 pfu SARS-CoV-2, which had not recovered their initial body weights by 25 dpi. Shown are peri-alveolar and intra-alveolar infiltrated areas building collagen fibers (blue).

bioRxiv preprint doi: https://doi.org/10.1101/2020.08.19.251249; this version posted January 29, 2021. The copyright holder for this preprint (which was not certified by peer review) is the author/funder. This article is a US Government work. It is not subject to copyright under 17 USC 105 and is also made available for use under a CC0 license.

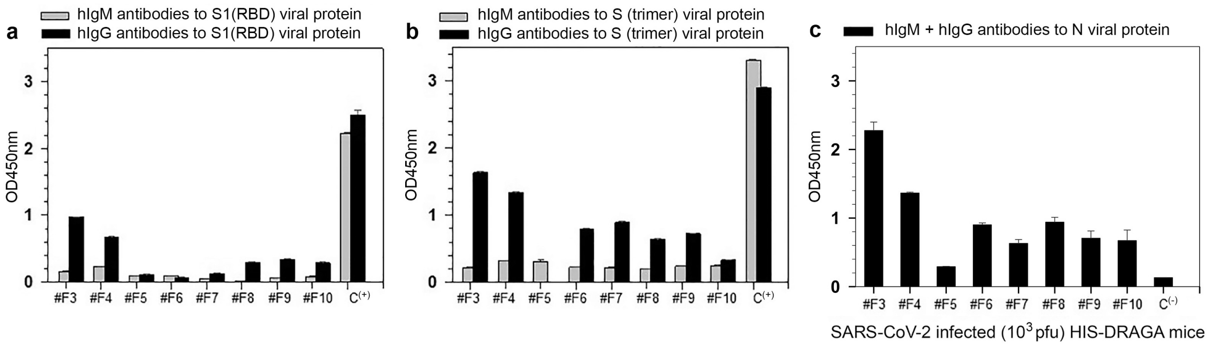

Fig. S12

Figure S12. **Antibody serum titers to SARS-CoV-2 viral proteins.** Titers of hIgM and hIgG antibodies to S1(RBD) protein (**a**) S-trimer protein (***b***) and N protein (**c)** in sera (diluted 1:20) from 8 HIS-DRAGA mice infected with SARS-CoV-2 (10^3 pfu), as measured by ELISA at 25 dpi. An anti-S1(RBD) antibody provided in the kit (Bethyl Laboratories) was included as a positive control in panels a and b (C+). The antibody titers against the N protein in serum from a non-infected mouse served as a negative control in panel c. OD450nm values were corrected by subtracting values (ranging from 0.045–0.067) of serum samples from the same mice prior to infection. Standard deviations (+/-SD) for each serum sample in duplicate wells were determined at 99% interval of confidence by SigmaPlot v.14 software.
